# Supplementary material for: The interactome of intact mitochondria by cross-linking mass spectrometry provides evidence for coexisting respiratory supercomplexes
Source: Mol Cell Proteomics. 2017 Dec 8;17(2):216–32. doi: 10.1074/mcp.RA117.000470 (PMC5795388; doi:10.1074/mcp.RA117.000470)
Supplement: Supplemental Data [file supp_RA117.000470_133922_0_supp_23978_fzffwf.zip › spectra_annotation/mito_DR_spectra_annotation/409-1-3-1-9-1.pdf]

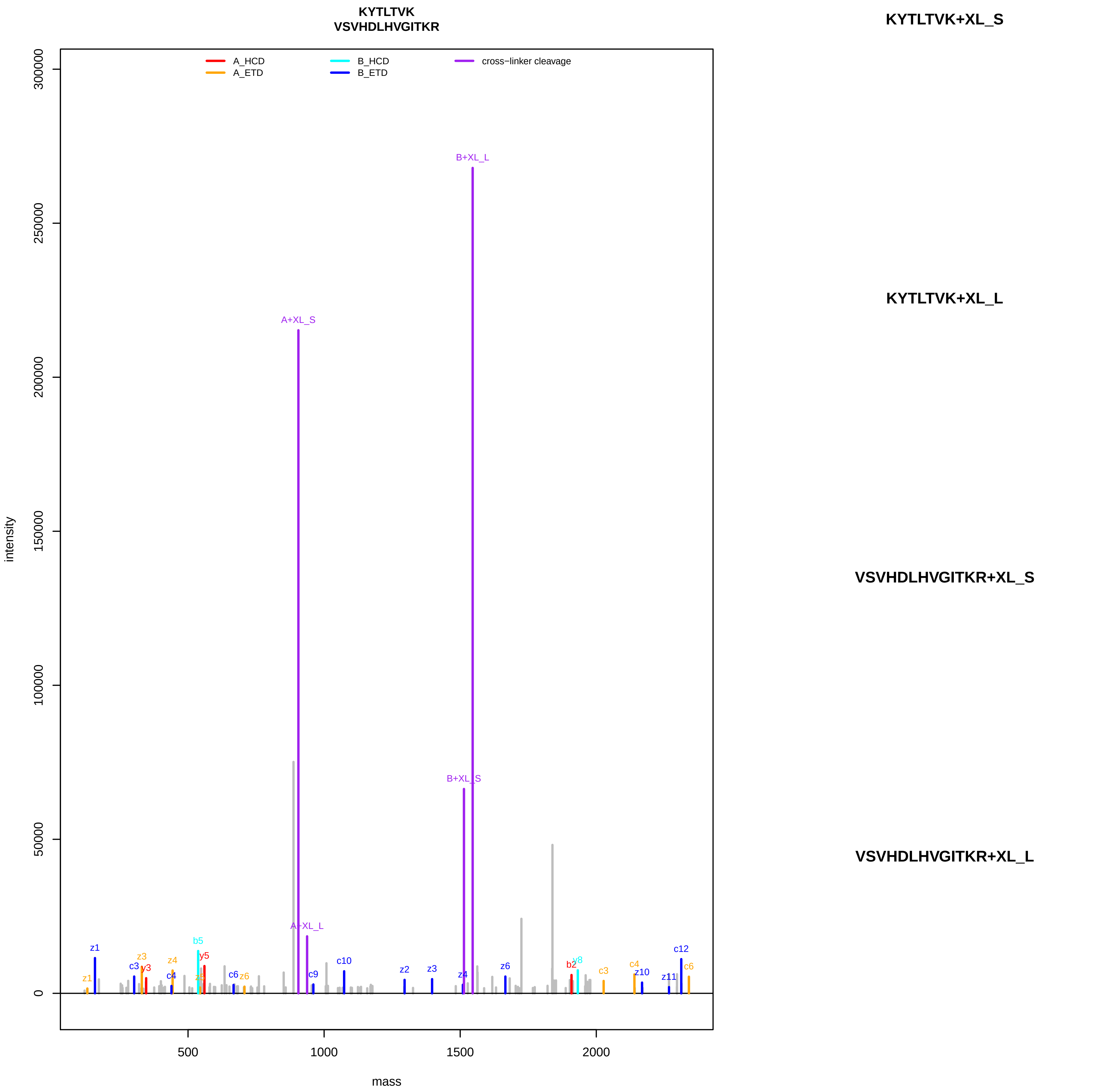
KYTLTVK
VSVHDLHVGITKR
A_HCD
A_ETD
B_HCD
B_ETD
cross−linker cleavage
A+XL_S
A+XL_L
B+XL_S
B+XL_L
z1
c3
z3
y3
c4
z4
b5
y5
c6
z6
c9
c10
z2
z3
z4
z6
b2
y8
c3
c4
z10
z11
c12
c6
intensity
mass
0
50000
100000
150000
200000
250000
300000
500
1000
1500
2000
KYTLTVK+XL_S
KYTLTVK+XL_L
VSVHDLHVGITKR+XL_S
VSVHDLHVGITKR+XL_L
